# Point-of-care ultrasound in cardiorespiratory arrest (POCUS-CA): narrative review article

**DOI:** 10.1186/s13089-021-00248-0

**Published:** 2021-12-02

**Authors:** Diana Ávila-Reyes, Andrés O. Acevedo-Cardona, José F. Gómez-González, David R. Echeverry-Piedrahita, Mateo Aguirre-Flórez, Adrian Giraldo-Diaconeasa

**Affiliations:** 1 Department of Critical Care Medicine, Universidad Tecnológica de Pereira, Grupo de Investigación Medicina Crítica Y Cuidados Intensivos (GIMCCI), Pereira, Colombia; 2 Department of Critical Care Medicine, Universidad Tecnológica de Pereira,, Pereira, Colombia; 3 Master en Ecocardiografía en Cuidados Intensivos, Sociedad Española de Imagen Cardíaca/Universidad Francisco de Vitoria, España, Pereira, Spain; 4 Department of Critical Care Medicine, Universidad Tecnológica de Pereira, Pereira, Colombia; 5 Grupo Investigación de Medicina Crítica Y Cuidados Intensivos (GIMCCI), Pereira, Colombia; 6 Department of Critical Care Medicine, Universidad Tecnológica de Pereira, Pereira, Colombia; 7 Department of Medicine, Universidad Tecnológica de Pereira, Pereira, Colombia

**Keywords:** Ultrasound, POCUS-point-of-care ultrasound, Cardiac arrest, Cardiopulmonary resuscitation, Echocardiography, Critical Care

## Abstract

The POCUS-CA (Point-of-care ultrasound in cardiac arrest) is a diagnostic tool in the Intensive Care Unit and Emergency Department setting. The literature indicates that in the patient in a cardiorespiratory arrest it can provide information of the etiology of the arrest in patients with non-defibrillable rhythms, assess the quality of compressions during cardiopulmonary resuscitation (CPR), and define prognosis of survival according to specific findings and, thus, assist the clinician in decision-making during resuscitation. This narrative review of the literature aims to expose the usefulness of ultrasound in the setting of cardiorespiratory arrest as a tool that allows making a rapid diagnosis and making decisions about reversible causes of this entity. More studies are needed to support the evidence to make ultrasound part of the resuscitation algorithms. Teamwork during cardiopulmonary resuscitation and the inclusion of ultrasound in a multidisciplinary approach is important to achieve a favorable clinical outcome.

**Supplementary Information:**

The online version contains supplementary material available at 10.1186/s13089-021-00248-0.

## Background

Cardiac arrest is the cessation of mechanical cardiac activity, as confirmed by the absence of circulation, and sudden cardiac arrest is an unexpected cardiac arrest that could result in attempts to restore circulation. If the attempts are unsuccessful, it is known as sudden cardiac death [1]. It is estimated that more than 356,000 out-of-hospital cardiac arrests occur annually in the United States of America. According to the American Heart Society's recent Heart and Stroke Statistics report, 90% of them are fatal [1]. After cardiac arrest, the survival at hospital discharge is approximately 10.4%, and survival with good functional status is 8.4% [2]. Around 209,000 in-hospital cardiac arrests occur in adults each year in the United States, with an average survival of 24.8% [3] and survival to discharge from hospital of 11.4% [3].

The mechanisms of cardiorespiratory a16 jrrest differ between out and in-hospital since in-hospital cardiac arrest is frequently the result of clinical deterioration, which typically occurs gradually for hours or days in the context of a critically ill patient. Cardiac arrest is frequently the result of noncardiac disorders of respiratory, hemodynamic, or neurological origin that cause a critical decrease in myocardial oxygenation, causing a reduction in contractility that culminates in pulseless electrical activity and ultimately asystole [4].

The basis for cardiac resuscitation includes the immediate provision of high-quality cardiopulmonary resuscitation combined with early defibrillation for defibrillable rhythms to impact the outcomes positively. These therapy bases lay the foundation for other possible interventions, such as medications, advanced airways, extracorporeal cardiopulmonary resuscitation, and post-cardiac arrest care, including targeted temperature control, cardiorespiratory support, and percutaneous coronary intervention [5]. Ultrasound is a diagnostic tool at the bedside of the patient that has been studied in the context of the patient in out and in-hospital arrest, and its importance has been suggested to be included in Advanced Cardiac Life Support (ACLS) algorithms [6–8].

The point of care ultrasound in cardiac arrest (POCUS-CA) conducted by a trained clinician allows the evaluation of the quality of the compressions, the quick diagnosis of reversible causes of arrest with non-defibrillable rhythms, the monitoring interventions, and its response to treatment. It also provides prognostic information regarding the possibility of a return to spontaneous circulation (ROSC) and survival [9].

During cardiopulmonary resuscitation (CPR), the POCUS-CA in the in-hospital setting, prioritizing the emergency department and the ICU, is a protocol to consider in the diagnosis of reversible causes of cardiac arrest in patients with non-defibrillable rhythms (pulseless electrical activity and asystole), such as cardiac tamponade, pulmonary embolism, tension pneumothorax, and hypovolemia [9], as well as to distinguish true asystole from fine ventricular fibrillation, conditions that have opposed therapeutic approaches. Current CPR guidelines recommend performing POCUS-CA when a reversible cause is suspected, although the impact on clinical outcomes is not yet clear [8, 10]. The literature mentions the importance of ultrasound in monitoring responses to interventions performed during resuscitation, real-time evaluation of the quality of compressions, and even as a guide to procedures such as decompression of tension pneumothorax and pericardiocentesis optimizing the safety and efficacy of the intervention [9, 11]. The most expert clinician must perform the POCUS-CA since the interpretation of images during compressions can be a challenge, and it entails the decisions to be taken in this context.

It is suggested that the POCUS-CA should be performed in the best possible window, ideally subxiphoid, and should not take more than 10 s, to minimize resuscitation interruptions [12, 13]. Given the importance that ultrasound has taken in this setting and its usefulness as a predictive tool, the importance of strengthening the available evidence through high-quality studies such as controlled clinical trials that allow integrating POCUS-CA into universal ACLS is highlighted.

## Ultrasound in cardiac arrest

Ultrasound has become a safe and accurate diagnostic tool for critically ill patients in different settings. Greater accessibility and portability have made ultrasound at the point of care an available tool for the clinician [14]. Initially, ultrasound opened a field in the intensive care unit to insert the vascular catheter guided by ultrasound [15–17]. Wider applications have now been established to improve critical care practice [18]. During brain-cardio-pulmonary resuscitation (CPR) the POCUS-CA, is a protocol to consider in the diagnosis of reversible causes of cardiac arrest in patients with non-defibrillable rhythms (Pulseless electrical activity and asystole), using a rational "holistic" approach, always starting with cardiac imaging (subxiphoid window and inferior vena cava, and if not sufficient, advance to the visualization of the parasternal long axis or apical four-chamber window) and then explore the next area, if necessary, based on cardiac imaging, with the clinical correlation [9]. In general, the cardiac ultrasound in arrest should be performed by the most expert ultrasound clinician to interpret the findings correctly, and its evaluation should not interfere with the development of resuscitation. The ultrasound provider must make a visualization of the subxiphoid window during compressions, since it is the window that offers a more affordable visualization, in addition to avoiding interruptions of cardiac compressions, and during the 10 s defined by the American Heart Association (AHA) algorithm in which pulse and rhythm are verified [5]. The other available windows should be evaluated to achieve timely diagnoses.

Two studies have concluded that the use of ultrasound in patients with cardiac arrest delays the restart of compressions and generates an increase in interruptions during resuscitation [19, 20]. In this sense, medical specialists must be intervened in ultrasound training to minimize the required time to obtain and interpret images in stressful situations [12]. To achieve this, for example, the implementation of checklists could be carried out to allow rapid image acquisition and control of interruptions [21].

### Technical aspects of the POCUS

POCUS-CA should ideally be performed with a probe appropriate to the expected objective. Lung evaluation requires a linear transducer with a 7–15 MHz frequency, which allows good resolution and low penetration. Cardiac evaluation requires a sectorial, low frequency (3.5–5 MHZ) transducer with high penetration for targeted assessment of structures. The convex transducer is preferred for the abdominal evaluation, which allows the assessment of deep tissues due to the high penetration, although with a lower resolution (frequency 2–5 MHz) [9]. POCUS-CA can be performed both transthoracic (TTE) and transesophageal (TEE). Using TTE, the clinician can assess the potentially reversible causes of cardiac arrest. By inserting the TEE probe into intubated patients, any reversible cause of arrest can be better elucidated, and the quality of chest compressions assessed, all while minimizing interruption of the compressions themselves. More studies are justified that evaluate echocardiography as a tool in cardiac arrest; however, TEE requires more training and level of expertise and presents certain limitations and risks [12].

### POCUS-CA objectives

#### Cardiac activity

The objective is the visualization of cardiac activity, defined as any visible movement of the myocardium, excluding the movement of blood within the cardiac chambers or the movement of the isolated valve (Additional file 2: Video S1, Additional file 3: Video S3). The POCUS-CA allows the differential diagnosis between asystole and fine ventricular fibrillation (VF).

When POCUS-CA is used, studies have shown that 10 to 35% of patients with asystole have a demonstrable cardiac contraction. The demonstration of cardiac contraction on initial ultrasound is of vital prognostic importance since it would indicate the possibility of returning to spontaneous circulation. It helps decision-making in terms of continuity of effort during CPR regardless of the time of resuscitation. It means chances of survival at discharge [9]. In a multicenter study published in the "Resuscitation Journal" in the United States in 2016 [11] "REASON STUDY" included 793 patients with out-of-hospital cardiac arrest who presented non-defibrillable rhythms, cardiac activity was present in 33% of the patients. It was associated with ROSC (OR 2.8, 1.9–4.2), survival at hospital admission (OR 3.6, 2.2–5.9), and survival at hospital discharge (OR 5.7, 1.5–21.9). In contrast, patients who lacked detectable cardiac activity had a poor ROSC and a lower overall survival rate. In general, the ROSC rate was greater than 50% if the cardiac activity was detected with ultrasound. It should be noted that the detection of ROSC is dependent on other variables and diagnostic tools. A meta-analysis that included eight studies, with a total of 568 patients with arrest, also showed a low probability of ROSC and survival when no cardiac activity was detected on ultrasound, but a modest increase in these results if cardiac contraction was present. In a study with 49 patients in the intensive care unit, it was concluded that 34.7% were in asystole and 65.3% in pulseless electrical activity (PEA); the ROSC rates were lower in those with electromechanical dissociation. Ultrasound performed during cardiopulmonary resuscitation in intensive care unit (ICU) patients can be performed without interfering with care protocols and can contribute to the differential diagnosis of arrest and the identification of a subgroup of patients with a better prognosis [22]. Although the ROSC and the overall survival rate are low when the cardiac contraction was not observed there is no conclusive evidence to define a standard criterion for decision-making about not starting or stopping resuscitation efforts; since there are still a low number of patients who can come out of the arrest; especially those with witnessed arrest, early CPR, short downtime, or a potentially reversible cause [23, 24].

#### Efficacy of cardiac compressions

Another objective is to evaluate the effectiveness of compressions by providing direct, real-time observation of compression and relaxation of the cardiac chambers during the cardiac massage. Adequate or high-quality compressions are associated with ROSC and survival. Literature reports improper hand position during resuscitation may lead to compression of the ascending aorta, aortic root, or outflow tract of the left ventricle, but not the left ventricle [25, 26]. By using the POCUS-CA, you can help adjust the applied forces and the location of the hands to optimize compressions [9].

The method for the proper evaluation of the quality of the compressions is transesophageal echocardiography (TEE). This technique requires advanced training to achieve adequate images that allow for timely interpretation, but it has the advantage that it does not interfere with resuscitation and therefore does not generate interruptions [12]. Constant visualization of the heart during compressions allows an objective assessment of resuscitation quality, determining the proper location of the hands of the provider of CPR to avoid obstruction of the left ventricular outflow tract and allowing adequate ventricular relaxation, which guarantees an adequate preload and cardiac output [27]. The real-time evaluation of cardiac contractility during TEE allows determining prognostic factors and the response to procedures such as cardioversion [28], in addition to providing better visualization of pericardial effusion, signs of tamponade, cardiac rupture, pulmonary embolism, valve disease, thrombi, cardiac masses, and the differentiation between true asystole and fine ventricular fibrillation (which have opposed therapeutic measures) [29].

## Practical approach use of the POCUS

**Fig. 1:**
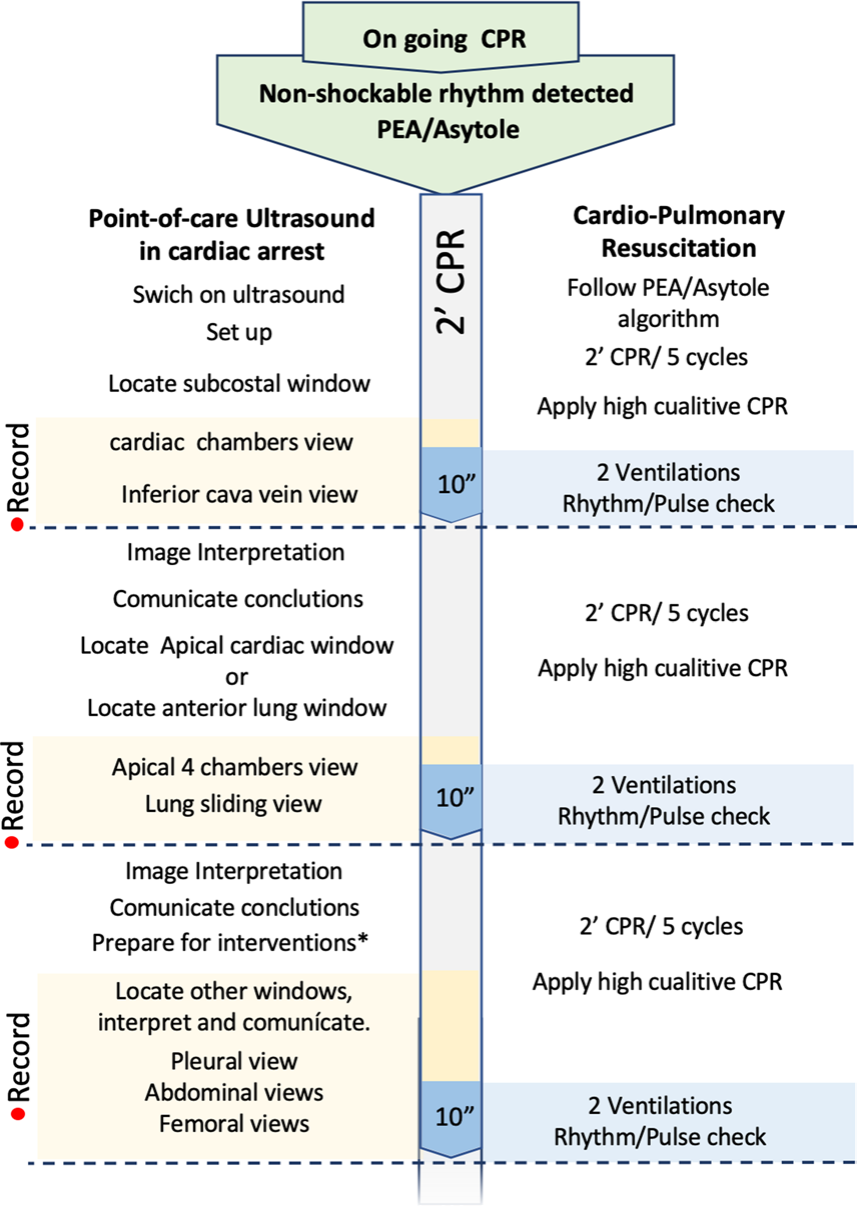
POCUS-CA Flowchart. *Echo-guided pericardiocentesis or thoracostomy.CPR: cardiopulmonary resuscitation, PEA: pulseless electrical activity

Cardiorespiratory arrest corresponds to a stressful situation for medical personnel, and all interventions must be carried out in an organized manner, including the use of ultrasound. The practical approach of the POCUS-CA in the in-hospital setting, prioritizing the emergency department and the ICU, is described in the flowchart (Fig. 1). Initially, it is necessary to make the gain and depth adjustments, evaluate the transducers available for evaluation, and start the engraving process according to the characteristics of the ultrasound scanner, since some devices may have a delay in starting the recording function and must be considered the memory available for the process.

**Fig. 2:**
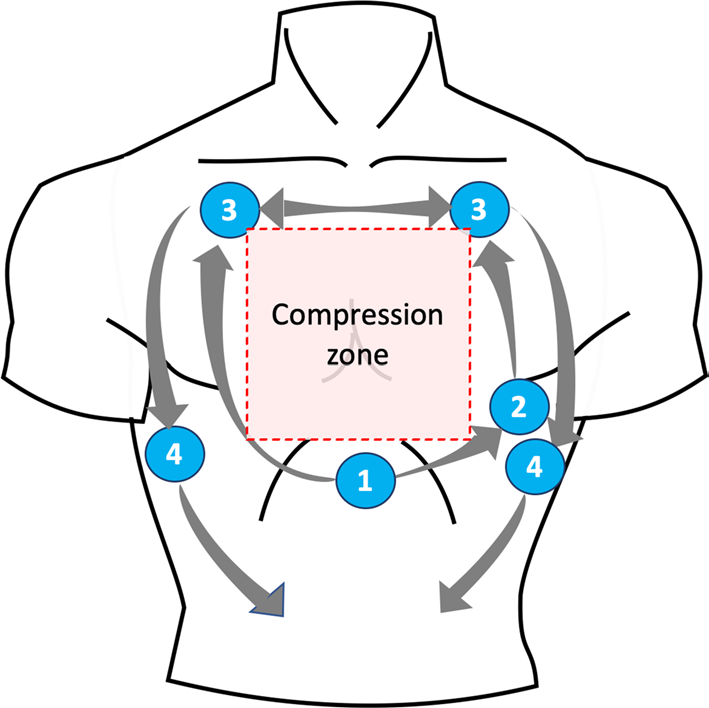
POCUS-CA Windows

Once the patient is in arrest, the first cycle of CPR should be allowed to be performed, and the rhythm to be defibrillable or non-defibrillable should be determined. During this cycle, the clinician in charge of performing the POCUS-CA prepares the equipment. Once the rhythm is described as non-defibrillable, it is recommended to start the POCUS-CA evaluation with the visualization of the subxiphoid window (subcostal 4 chambers) and obtain the image's recording. The evaluation of the images is not carried out during direct visualization, since this may delay the restart of the compressions, that means that the recording will be carried out for 10 s as indicated in the international resuscitation guidelines, and when the compressions are restarted, the clinician will evaluate the recording obtained to determine diagnostic findings, the findings are communicated and joint decision-making with the CPR team is carried out. The window used for assessing the patient with arrest during resuscitation is the subxiphoid window as the first option and the apical four chambers as the second option since they do not intervene with compressions (Fig. 2). During the pause for pulse taking and rhythm evaluation, the other windows available for diagnosis can be visualized, for a limit of 10 s, which are the pulmonary window in the assessment of pleural glide, the 4-chamber apical cardiac window, parasternal short axis, and as the last option the parasternal long axis. Windows can be used to evaluate pleural effusion, hemothorax, and free intra-abdominal fluid, described in the Extended Focused Assessment with Sonography in Trauma (FAST-E) protocol [30], which does not require synchronization with the CPR, and allow obtaining additional information on the patient's diagnosis. The POCUS-CA allows the determination of potentially reversible causes of arrest and should be integrated to the "5H's and 5 T's" checklist of cardiac arrest management (The **H's and T's** of ACLS; hypovolemia, cardiac tamponade, pneumothorax, and pulmonary embolism (Table 1) [8, 9, 12].

**Table 1:** 5H's and 5 T's" checklist [8]

	H's	T's
Cardiac arrest	Hypovolemia^*us*^ Hypothermia Hypoxia Hypo or hyperkalemia Hydrogen ion (acidosis)	-Cardiac tamponade^*us*^ -Toxins -Tension pneumothorax^*us*^ - Pulmonary thrombosis^*us*^ Thrombosis coronary

*US* ultrasound

### Diagnosis of reversible causes of cardiac arrest

The diagnosis of reversible causes allows early decision-making and could have a considerable impact on survival [12]. There are several echocardiography protocols in a patient with cardiac arrest. The Focus-Assessed transthoracic echocardiography (FATE) protocol, published by Jensen et al., was performed on 210 patients in 20 intensive care units and evaluated the subcostal, apical, parasternal, and pleural windows [31]. In 2005, the rapid cardiac ultrasound was developed by clinicians with basic training, which only includes the subxiphoid window for evaluation of primary findings [32]. The Focused echocardiographic evaluation in resuscitation management (FEER) protocol (FEEL) published by Breitkreutz et al., evaluated the subcostal, parasternal and apical window, which showed an improvement in the results [33], and later Prosen et al. published a modification of the FEER protocol [34]. The Cardiac arrest ultra-sound exam (CAUSE) [35] protocol published in 2008 evaluates the subcostal or parasternal or apical window added to the evaluation of the pulmonary window and has been widely disseminated in clinical practice. Testa's proposal of an integrated ultrasonographic approach into the ALS algorithm for cardiac arrest (PEA) protocol includes epigastric, pulmonary, and abdominal evaluation during CPR [36].

Dr. Lichtenstein proposes the Sequential Echographic Scanning Assessing Mechanism Or Origin of Severe Shock of Indistinct Cause (SESAME) protocol and suggests starting with a lung scan to rule out possible causes leading to cardiac arrest. First, pneumothorax must be ruled out. Second, a partial diagnosis of pulmonary embolism is made following the bedside lung ultrasonography (BLUE) protocol. Third, fluid therapy can be guided following the Fluid Administration Limited by Lung Sonography (FALLS) protocol. The SESAME protocol continues to scan the lower femoral veins to check for signs of deep vein thrombosis (DVT), followed by examining the abdomen for massive bleeding. The pericardium is then evaluated to exclude pericardial tamponade. Finally, a transthoracic cardiac ultrasound is performed to verify other (cardiac) causes that lead to cardiac arrest. The emphasis is on a holistic approach, where ultrasound can be the modern stethoscope to complete the physiological examination of critically unstable patients [37]. In 2017, a consensus of an expert panel was published and recommended the cardiac arrest protocol (CORE) at the time of POCUS and in specific clinical questions.

Central views are limited to cardiac views and should be performed during the rhythm check pause in chest compressions without causing a prolonged interruption in chest compressions. The best views are the subxiphoid (SUBX) and the apical four chambers (A4C), but the long axis parasternal window can be used if necessary. Other views include pulmonary windows (for the absence of lung sliding in pneumothorax or pleural fluid) and subcostal window for evaluation of the inferior vena cava (IVC). Either view should be used to search for pericardial fluid and examine ventricular shape (e.g., right heart pressure) and cardiac function (e.g., asystole versus organized cardiac activity).

Additional applications that may help during cardiac arrest include using POCUS for endotracheal tube confirmation, scanning of proximal leg veins for deep vein thrombosis (DVT), or searching for sources of blood loss (aortic artery evaluation, peritoneal or pelvic fluid).

These protocols represent a consensus on the prioritization of the examination for these critically ill patients, based on the probability of detecting the underlying pathology and the perceived impact on the patient's treatment [38].

#### Cardiac tamponade

Cardiac tamponade is one of the potentially reversible causes that can lead to cardiac arrest. Among the etiologies, they are known to be traumatic and non-traumatic in origin and, in general terms, acute pericardial effusions as small as 50 ml can cause tamponade. In contrast, under chronic conditions, the pericardium can slowly stretch to accommodate large effusions over time without causing tamponade. The most relevant echocardiographic findings that guide us towards cardiac tamponade are the presence of pericardial effusion visible in all windows, but the ideal ones are the subxiphoid, apical, and parasternal long axis, and it is relatively easy to recognize [39]. Pericardial effusion is generally recognized by a black or anechoic appearance (Additional file 4: Video S4). However, effusions resulting from an inflammatory or infectious condition may have a lighter or more echogenic gray appearance [40]. The traumatic pericardial effusion acquires a more hyperechoic appearance. To visualize the effusion, the reclining patient should be located as far as possible. In this position, the smallest effusions will be found in the posterior layer of the heart. As the effusions grow, they will surround the heart circumferentially and move into the anterior pericardial space [41]. Most effusions will flow freely into the pericardial sac, although loculated effusions may occasionally occur. Loculated or septate effusions generally occur in patients who have undergone cardiac surgery and in inflammatory conditions [39]. Spills are classified as minor, moderate, or large according to the following scale [40].

- Minor: Less than 1 cm deep, non-circumferential around the heart.
- Moderate: Less than 1 cm deep, circumferential around the heart.
- Large: More than 1 cm deep, circumferential around the heart.

It is essential to know how to differentiate pericardial effusion from pleural effusion; the pericardial effusion will be in front of the descending aorta and above the posterior pericardial reflection in the parasternal long axis view. In contrast, pleural effusions will be located behind the descending aorta and below posterior pericardial reflection [40]. The pericardial or epicardial fat pad can sometimes be mistaken for a pericardial effusion. The specific location of this structure is in the area just before the heart and below the anterior or near-field pericardial reflex, which is located within the pericardial sac. The pad has a classic appearance, with shiny or hyperechoic regions. In the parasternal window, an isolated anterior location suggests more a fat pad than an effusion. For an effusion to be visualized only on the anterior side of the pericardial sac, a circumferential effusion would generally be present. A downside to subxiphoid vision is that ascites can sometimes be mistaken for a pericardial effusion. Differentiation between the two is possible by noting that ascites will be located closer to the probe surrounding the liver within the abdominal cavity and outside the pericardial sac. A pericardial effusion will be found within the pericardial sac, below pericardial reflection near field and adjacent to the heart [40].

As mentioned, the presence of pericardial effusion does not make a diagnosis of cardiac tamponade [40], a competition of additional findings is required such as right atrial systolic collapse and right diastolic ventricular collapse (Additional file 5: Video S5). However, these signs are not seen in the patient with arrest, but they can occur prior to the arrest. In the patient before the arrest, the experts describe energetic cardiac movements as the "furiously contracting chamber," with hyperdynamic atrial contractions and exaggerated movements as tamponade progresses, making it challenging to assess systolic contraction for diastolic collapse (Additional file 1: Video S1). Although both right heart chambers must be evaluated, the diastolic collapse of the right ventricle is a more specific finding. This collapse is best understood as a spectrum of ultrasound findings of a subtle sudden deviation of the wall in diastole to complete compression of the chamber. In patients with pulmonary hypertension, the diastolic collapse of the right heart is a very late finding, so if you are in the context of a patient without underlying pathology, you must think of acute symptoms.

**Fig. 3:**
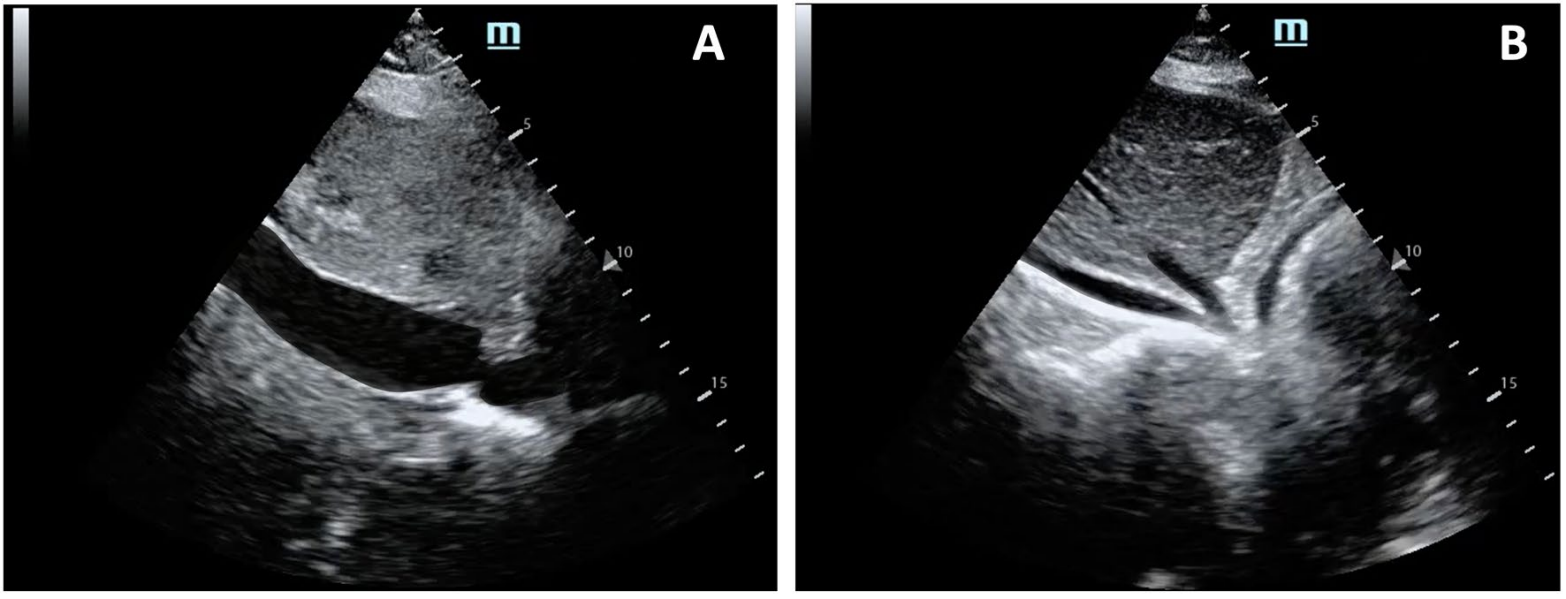
IVC view: A. Dilated (Video 06), B. Flattened (Video11)

**Fig. 4:**
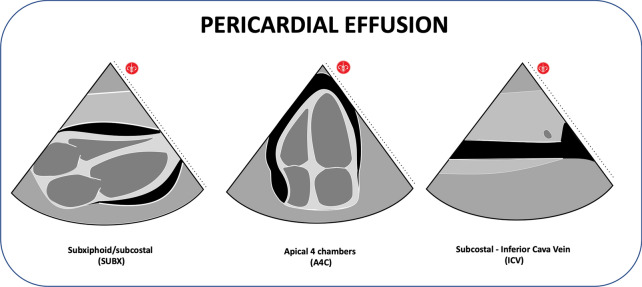
Pericardial effusion and Cardiac tamponade findings

The inferior vena cava (IVC) can also be evaluated, which, if found plethoric, could indicate the increase in the atrial cavities (Fig. 3; Additional file 6: Video S6). These findings have a sensitivity of 96% and a specificity of 98% [40] (Fig. 4). Another more advanced finding includes changes in transvalvular flow velocity on the Doppler, which requires more training and evaluation time, which in the context of CPR may not be applicable, but if once the patient is evaluated at a stage of ROSC and hemodynamic stability [40].

In the patient with cardiac arrest presenting signs compatible with cardiac tamponade [41]^.^ [42], ultrasound-guided pericardiocentesis can be performed emergently [43].

#### Pulmonary thromboembolism (PE)

**Fig. 5:**
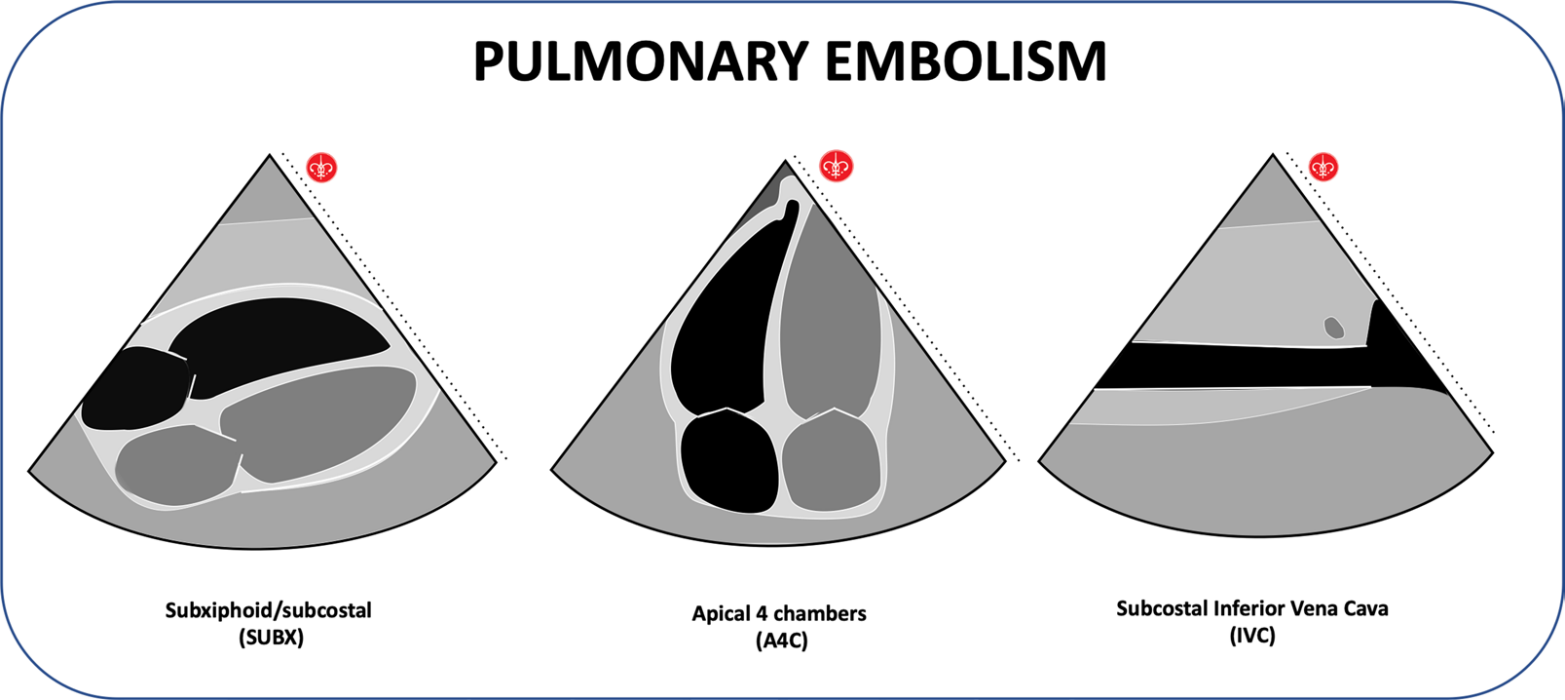
Pulmonary embolism findings

Massive pulmonary thromboembolism can be detected by ultrasound with direct and indirect signs. The direct sign is the visualization of the clot, and the indirect ones include the acute dilation of the right ventricle (RV), exceeding the normal relationship with the left ventricle (LV), which must be 0.6: 1 in the apical four-chamber view, and that for it to be diagnostic of RV dilation should be a 1: 1 ratio Additional file 7: Video S7. The finding of the D-shaped RV in the parasternal short-axis window is another of the signs of RV dilatation [44]. In the pre-arrest patient, the deviation of the ventricular septum to the left side, paradoxical systolic septal movement, septal flattening in diastole, McConnell sign (although not exclusive to PE) can be seen [45] (Fig. 5; Additional file 8: Video S8). These ultrasound findings confirm the right ventricular tension that can be observed in a severe pulmonary embolism. Right strain should be differentiated from chronic right heart pressure, which is generally seen in conditions of long-standing pulmonary arterial hypertension (such as primary pulmonary hypertension and cases of chronic pulmonary embolism); the right ventricular wall will compensate by hypertrophy, often larger than 5 mm. In acute right ventricular tension, the chamber will not have time to compensate for hypertrophy, and typically a thin wall measuring less than 5 mm will be seen [39]. The prominent trabecular architecture of the chamber and papillary muscles can also be recognized. When asked if the right ventricular chamber dilation is acute or chronic, the clinician can use these general rules to decide whether more emergent therapy, such as fibrinolysis, may be indicated. As previously mentioned, some protocols also include ultrasound signs of intraluminal thrombus in the deep veins" of the lower limbs for the diagnosis of pulmonary embolism (PE) during arrest [37]. The approach to confirm the diagnosis of deep venous thrombosis can be done more elaborately and more comprehensively (common femoral vein, calf veins, upper extremities) [37].

#### Tension pneumothorax

**Fig. 6:**
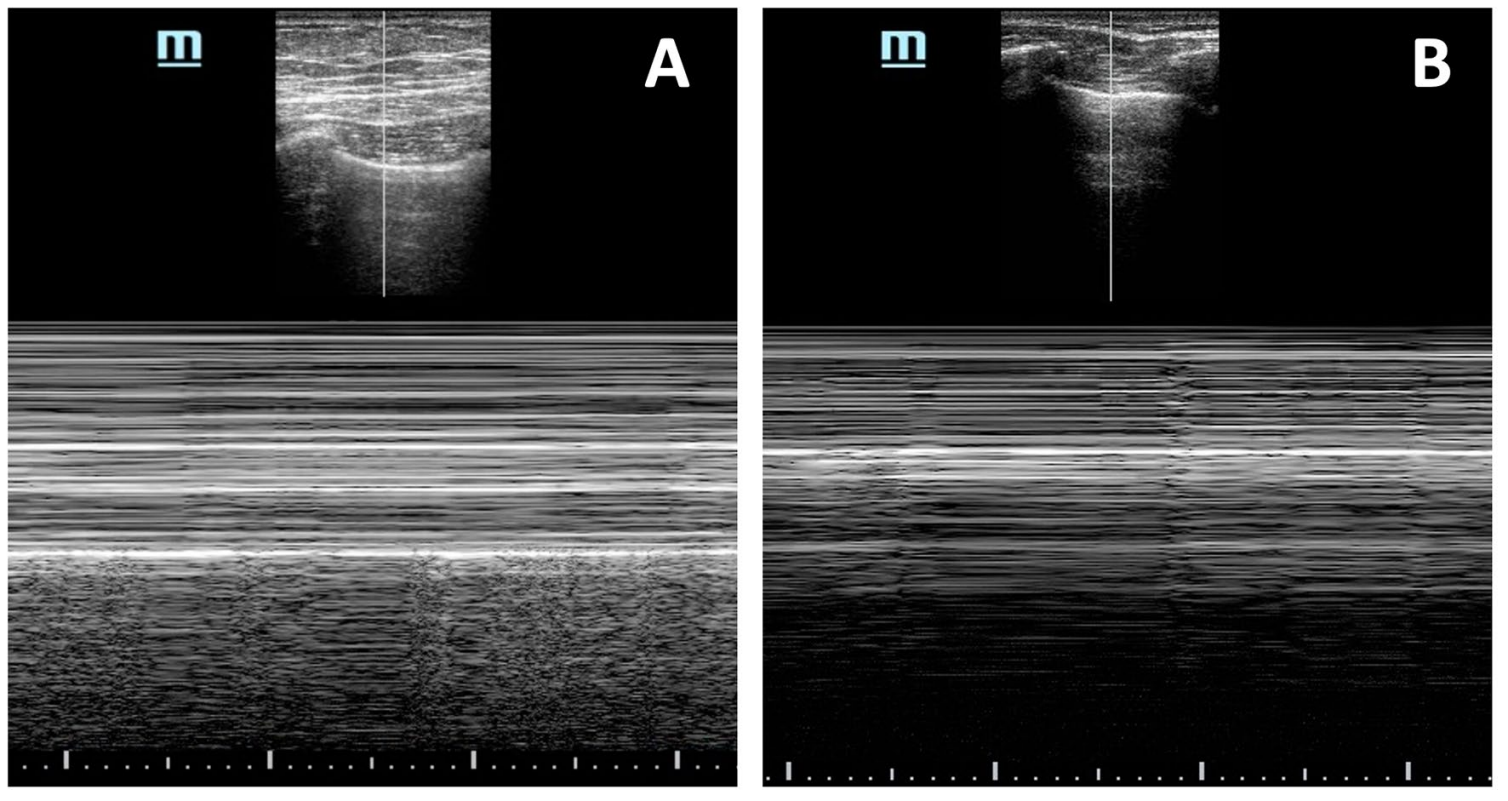
Anterior pulmonary window (M mode). A) beach sign b) code bar sing

Tension pneumothorax is one of the leading causes of potentially reversible arrest in patients with traumatic etiology [46]. Acute decompression of pneumothorax is imperative to improve the patient's ventilatory mechanics and hemodynamics. Usually, the physical examination of tension pneumothorax may not be problematic in this context; however, in critically ill patients with mechanical ventilation, it may not be so easy to suspect [47]. Ultrasonographic findings of pneumothorax are visualized in the 4–5 intercostal space with a mid-clavicular line; they are the absence of pleural sliding, the sign of the stratosphere or barcode, the lack of B lines, and absence of a pulmonary pulse (Fig. 6). The lung point is the only sign that confirms pneumothorax, with very high specificity. However, extending the examination to the whole chest during a critical condition may be difficult.

**Fig. 7:**
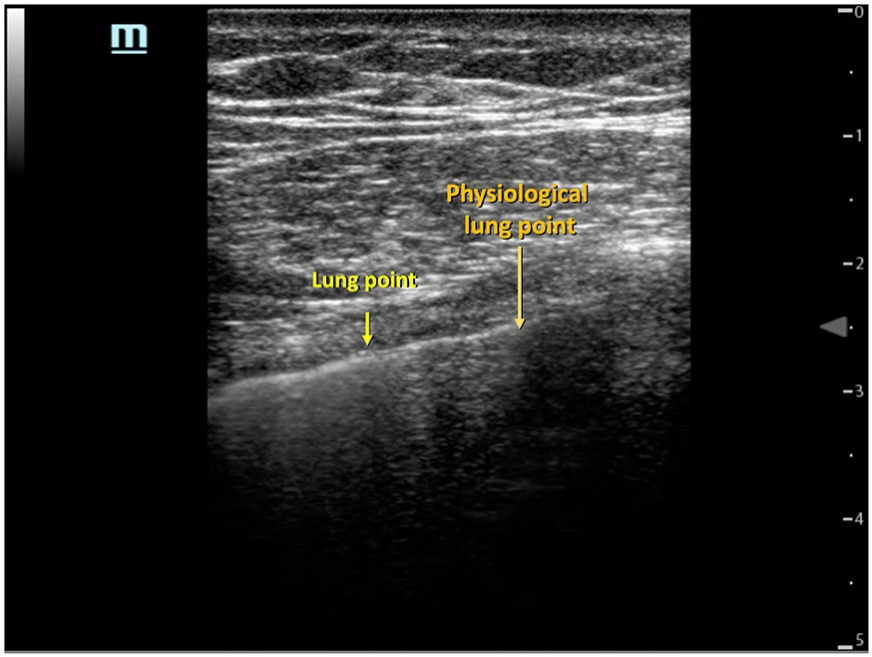
Lung point sign and mediastinal point sign (Video 08)

Moreover, when the lung is totally collapsed, the lung point will be absent. The combination of the absence of sliding B-lines and pulse has the optimal sensitivity in ruling out pneumothorax. Their specificity is also high, but only in the critical scenario of a cardiac arrest [48]. A frequent pitfall is misunderstanding the physiological lung point sign or "mediastinal point sign" as if it were a pathological lung point. The mediastinal point sign is found in the 4th–5th intercostal space with the midclavicular line of the left hemithorax and is characterized by the lung sliding absence but with the underlying cardiac movements instead of the pulmonary A-line pattern and differs from the lung point sign that shows lung sliding absence but with A-line pattern and lack of another lung artifact sings (e.g., lung pulse, B lines) [49] (Fig. 7; Additional file 9: Video S9). Also, ultrasound can safely guide acute decompression of pneumotorax [40].

Hemothorax is another finding of the trauma patient, and if it is massive, it could generate hypovolemic shock, circulatory and pulmonary collapse. Ultrasonography can assess the volume and nature of effusion in addition to indicating the appropriate area for thoracentesis and thereby decrease the risk of complications. The volume calculation, in general, is done using the distance formula mm X 20; although this has not been validated in the patient with arrest, it could be considered once the patient stabilizes to define the need for intervention.

#### Hypovolemia

**Fig. 8:**
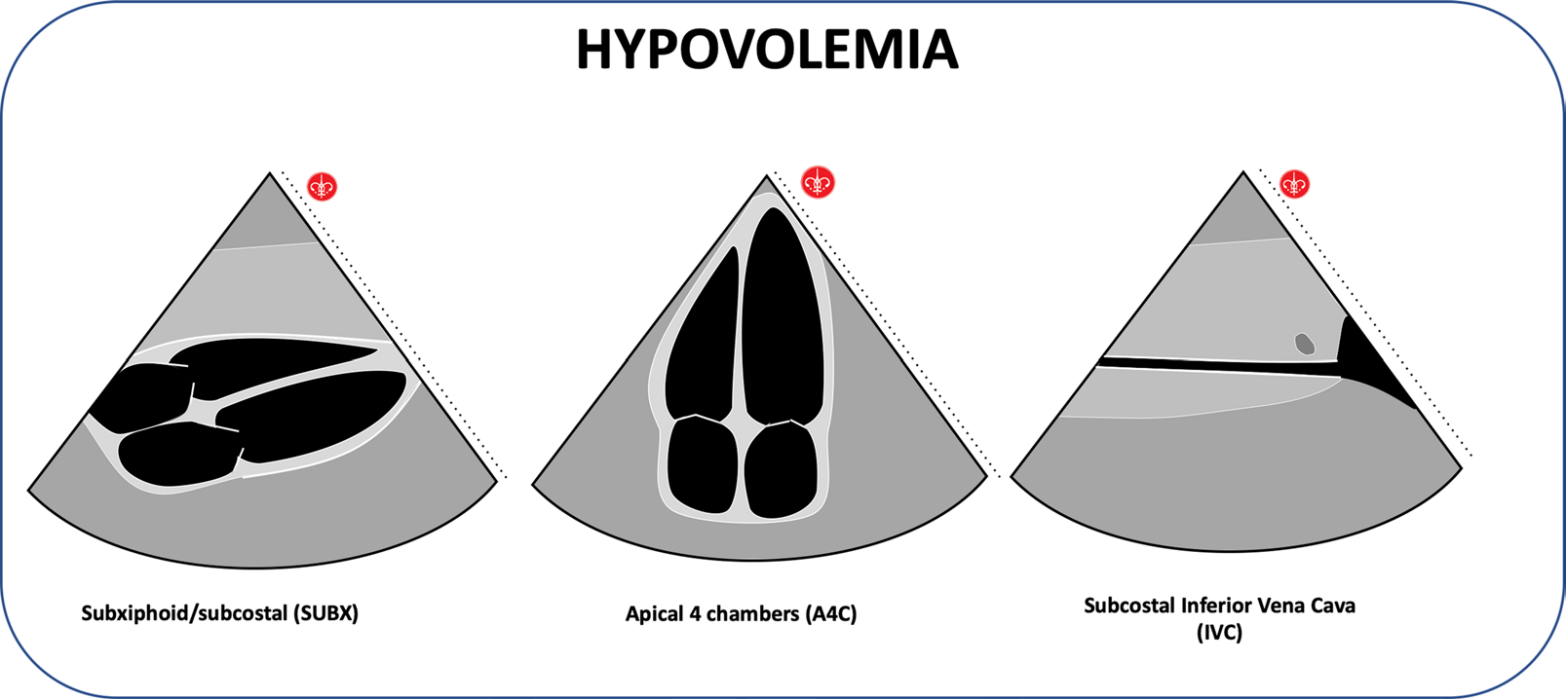
Hypovolemia findings

The shock patient can rapidly progress to circulatory collapse. In the context of the trauma patient, the hemorrhagic hypovolemic shock should be considered initially, and the cardiogenic, distributive, or obstructive shock [50] should be considered in the medical patient, each profile with a different echocardiographic finding [51, 52], which can be evaluated using protocols such as RUSH [53, 54]. Hypovolemia in the patient with an pre-cardiac arrest can be assessed using sharable ultrasound findings with accelerated movements, hyperdynamic pattern, obliteration of the ventricles, small ventricular area at the end of systole and diastole (Additional file 10: Video S10; Additional file 11: Video S11), "kiss sign" in the Parasternal Short View, which is nothing more than the union of the ventricular walls and the papillary muscles in each beat due to the lack of intracavitary volume. The evaluation of the inferior vena cava (IVC) with the sign of "Empty tank" (diameter less than 2 cm, IVC collapse > 50% during inspiration); compatible with central venous pressure (PVC < 10cmH2O), is another of the signs that indicate hypovolemia [40]. In the patient with arrest, it can be visualized the obliteration of the ventricles and the collapsed inferior vena cava (IVC diameter < 2 cm), which added to the clinical course and other findings make us think of hypovolemia [53] (Fig. 8; Additional file 12: Video 12).

The RUSH protocol [53] allows an initial approach to the patient in shock by addressing three main parameters described as "pump, tank, and pipes", allowing the classification of the type of shock that the patient has according to the findings in each of the parameters, and facilitating the Etiological diagnosis of the clinical condition at the bedside in a fast and comprehensive way. The protocol simplifies the ultrasound evaluation into the physiological paradigm of "pump, tank, and pipes," allowing the clinician to remember the critical aspects of the exam components easily [55]. Although the RUSH protocol is helpful, it has limitations because of the lack of precision in the definitions of the findings and diagnostic patterns. In this sense, Volpicelli [56] et al. published a study in 2013 that enrolled 108 adult patients complaining of non-traumatic symptomatic hypotension of uncertain etiology. Patients received immediate point-of-care ultrasonography to determine the cardiac function and right/left ventricle diameter rate, inferior vena cava diameter, and collapsibility, pulmonary congestion, consolidations and sliding, as well as abdominal free fluid, aortic aneurysm, and deep venous thrombosis. The organ-oriented diagnoses were combined to formulate an ultrasound hypothesis of the cause of the hemodynamic instability. The ultrasound diagnosis was then compared with a final clinical diagnosis, obtaining as a result an adequate concordance between the point-of-care ultrasonography diagnosis and the definitive clinical diagnosis. In summary, the integration of a multiorgan ultrasonographic protocol in the diagnostic process of undifferentiated hypotension has great potential in guiding the first-line therapeutic approach [56].

Ultrasound allows the evaluation of signs of shock in the patient in pre-arrest and its evaluation during the post-arrest period to make etiological diagnoses that allow therapeutic decisions to be made once the patient is stable [12], for example, in the case of an acute myocardial infarction, a complete evaluation can be carried out on the left ventricular ejection fraction (LVEF), contractility disorders and anatomy of the papillary muscles to help determine the need for emergent cardiac catheterization, and in the case of massive pulmonary thromboembolism findings may guide thrombolysis. Ultrasound allows real-time evaluation of the response to interventions and allows to guide diagnostic procedures. The Ultrasound signs are found in (Table 2).

**Table 2:** Ultrasound signs for reversible causes

	View	Arrest signs	Peri-Arrest signs
Cardiac Tamponade	Subxiphoid View Apical 4 chambers Subcostal IVC Parasternal Long View*	Pericardial effusion Plethoric IVC	Pericardial effusion Plethoric IVC Systolic collapse of the right atrium Collapse of the right ventricle in diastole
Hypovolemia	Subxiphoid View Apical 4 chambers Subcostal IVC Parasternal views*	Obliteration of ventricles Inferior vena cava diameter < 2 cm	Hyperdynamic Obliteration of ventricles Kiss sign "Empty tank" inferior vena cava (IVC diameter < 2 cm collapse > 50% during inspiration)
Pulmonary Embolism	Subxiphoid View Apical 4 chambers Subcostal IVC Parasternal views* Lower limb compression ultrasound*	Right heart thrombus Right ventricular dilation D sign of left ventricle Deep vein thrombosis	Pulmonary Hypertension Deep vein thrombosis Paradoxical septal systolic movement Septal flattening in diastole McConnell sign D sign of left ventricle
Tension pneumothorax	4th–5th intercostal space with midclavicular line	Absence lung sliding (during artificial ventilation) Stratosphere /barcode sign	Absence lung sliding Stratosphere /barcode sign Absence B lines Absence pulmonary pulse Lung point

*Optional

#### Evidence of ultrasound in arrest as a prognostic tool

Ultrasound in arrest has been studied in intrahospital and outpatient settings. As mentioned, there are multiple protocols to carry out the POCUS-CA, which require more extensive external validation, and so far, there is no study indicating which is superior; however, it is clear the more windows that are evaluated, the greater the success in collecting findings to integrate the information and make an adequate clinical interpretation, essential for decision-making that can modify the patient's prognostic course [17]. The POCUS-CA approach requires training, and some studies have concluded that the use of ultrasound in this setting can prolong the interruptions and the restart time of CPR and thus worsen the results. In 2018, a prospective study was published of 276 patients who underwent the CASA protocol (Cardiac Arrest Sonographic Assessment), a protocol that evaluates three main questions between each CPR cycle; cardiac tamponade, RV stress, and cardiac activity, with yes / no responses, and lasting less than 10 s. The protocol was carried out by residents of the university institution, and within the results, they found a reduction in the time of interruptions of 19.8–15.8s [57]. In 2019 the SHoC-ED group published a retrospective cohort study of 180 patients undergoing POCUS-CA; it concluded that POCUS-CA emergency department patients received more extended resuscitation with higher intervention rates than those with negative findings or when POCUS-CA was not performed. Patients with cardiac activity in POCUS-CA had improved clinical outcomes compared to patients who did not receive POCUS-CA and patients without activity in POCUS-CA [58]. A meta-analysis published in Resuscitation [59] of 15 studies with a total of 1695 patients concluded that POCUS-CA could be used to identify reversible causes and predict short-term outcomes in patients with cardiac arrest. In patients with a low prior probability of ROSC, the absence of spontaneous cardiac movements on echocardiography can predict a low chance of survival and guide the decision to end resuscitation. Another systematic review and meta-analysis of 10 studies with 1486 patients confirmed that cardiac activity in POCUS-CA was associated with better probabilities of ROSC and survival at discharge, in non-traumatic cardiac arrest, and with non-defibrillable rhythm [60]. The overall conclusion of the evidence is that the evaluation of cardiac motion on transthoracic echocardiography is a valuable tool in predicting short-term cardiac resuscitation outcomes. Given the safety and availability of ultrasound in the emergency department, it is reasonable to apply POCUS-CA to cardiopulmonary resuscitation if its use does not interrupt resuscitation [61]. Table 3 shows the different protocols described in the literature.

**Table 3:** Ultrasound protocols in cardiorespiratory arrest

2004. Jensen et al. [31]	FATE	1. Subcostal view 2. Apical view 3. Parasternal view 4. Pleural view
2005. Niendorff et al. [32]	Rapid Cardiac Ultrasound	1. Subcostal view
2007. Breitkreutz et al. [33]	FEER (FEEL)	1. Subcostal view 2. Parasternal view 3. Apical view
2008 Hernandez. [35]	CAUSE	1. Subcostal view or apical view or parasternal view 2. Lung view
2010 Prosen et al. [34]	Modified FEER	1. Subcostal view 2. Parasternal view 3. Apical view
2010 Testa et al. [36]	PEA	1. Epigastric scan 2. Pulmonary scan 3. Abdominal scan
2015. Lichtenstein. [37]	SESAME	1. Pulmonary view 2. Cardiac view 3. DVT detection at the V-point
2017 Arkinson et al. [38]	CORE	Consensus on the use of point of care ultrasound for undifferentiated hypotension and during cardiac arrest
2019 Arkinson et al. [56]	SHoc-ED	Sonography in Hypotension and Cardiac Arrest in the Emergency Department
2021. Ávila, Acevedo et al	POCUS-CA	1. Subcostal view 2. Apical view 3. Parasternal view 4. Pleural and pulmonar view 5. FAST-E

CPR techniques have evolved in the last decade in terms of implementing high-quality measures, early intervention in defibrillable rhythms for the impact of the electrical phase of arrest [62]. Still, it continues to have unsuccessful results given the low rate and survival, influenced by multiple factors. Mechanical ventilation has physiological effects on the patient in cardiac arrest, and thus clinical challenges are posed on the mechanical ventilation strategy to be implemented during the arrest. Although there is still a lack of conclusive scientific evidence on the ventilatory mode and the parameters to be programmed in the ventilator during compressions in resuscitation, it is imperative further studies to determine the effects that mechanical ventilation has on ultrasound findings during cardiopulmonary resuscitation [63].

The intensivist involved may have information on the physiological conditions that led to the patient's arrest, but on some occasions, particularly in the emergency department patient, there is little information on the reason for cardiac arrest and the probability of survival. In this setting, bedside ultrasound shows promise as a critical tool in providing valuable information to help direct management. It is important to remember that the decision to end resuscitation should never be made based on ultrasound findings alone [21]. The POCUS in cardiac arrest is an excellent complementary tool in the hands of experienced providers. Although no current studies show an improvement in mortality, echocardiography is useful for diagnostic and prognostic purposes in cardiac arrest [12].

The cardiac activity present in the POCUS-CA is a finding of prognostic importance for ROSC and survival, as evidenced in multiple studies [23, 24, 64–76].

## Conclusions

Ultrasound has become a safe and accurate diagnostic tool for critically ill patients in the intrahospital setting, prioritizing the emergency department and the ICU. Greater accessibility and portability have made ultrasound at the point of care an available tool for the intensivist [14]. The POCUS-CA in the hands of a trained clinician, allows the evaluation of the quality of the compressions, the rapid diagnosis of reversible causes of arrest with non-defibrillable rhythms, the monitoring of interventions in terms of response to treatment, and provides prognostic information regarding the possibility of ROSC and survival [9]. Given the importance that ultrasound has taken in this setting and its usefulness as a predictive tool, the importance of strengthening the available evidence through high-quality studies such as controlled clinical trials that allow integrating POCUS-CA into universal CPR algorithms is highlighted. In this sense, medical specialists must be intervened in ultrasound training to minimize the time necessary to obtain and interpret images in stressful situations [12]; the implementation of checklists could be carried out to allow rapid image acquisition and control of interruptions. [21].

## Supplementary Information

**Additional file 1: Video S1.** SUBX Pericardial efussion, right ventricule colapse.

**Additional file 2: Video S2.** PEA -Cardiac Activity.

**Additional file 3: Video S3.** PEA_Asytole non cardiac activity- dilated ICV.

**Additional file 4: Video S4.** Pericardial efussion A4C.

**Additional file 5: Video S5.** Pericardial efussion, right ventricule colapse SUBX.

**Additional file 6: Video S6.** Dilated ICV.

**Additional file 7: Video S7.** Pulmonar embolism—SUBX.

**Additional file 8: Video S8.** Pulmonar embolism—McConnell sign A4C.

**Additional file 9: Video S9.** Lung point and pelural poinrt (physiological lung point).

**Additional file 10: Video S10.** Hyperdinamic_ hypovolemic SUBX.

**Additional file 11: Video S11.** Hyperdinamic_ hypovolemic A4C.

**Additional file 12: Video S12.** Flattened ICV.

## Data Availability

Supplemental digital content.

## Abbreviations

ACLS: Advanced cardiac life support
POCUS-CA: Point of care ultrasound in cardiac arrest
ROSC: Return to spontaneous circulation
CPR: Cardiopulmonary resuscitation
AHA: American Heart Association
TTE: Transthoracic echography
TEE: Transesophageal echography
VF: Ventricular fibrillation
PEA: Pulseless electrical activity
ICU: Intensive care unit
FAST-E: Extended Focused Assessment with Sonography in Trauma
FATE: Focus-Assessed transthoracic echocardiography
FEER: Focused echocardiographic evaluation in resuscitation management
CAUSE: Cardiac arrest ultra-sound exam
PEA: The proposal of an integrated ultrasonographic approach into the ALS algorithm for cardiac arrest
SESAME: Sequential Echographic Scanning Assessing Mechanism Or Origin of Severe Shock of Indistinct Cause
BLUE: The bedside lung ultrasonography
FALLS: Fluid Administration Limited by Lung Sonography
DVT: Deep vein thrombosis
CORE: Cardiac arrest protocol
SUBX: Subxiphoid
A4C: Apical 4 chambers
IVC: Inferior vena cava
PE: Pulmonary thromboembolism
RV: Right ventricle
LVEF: Left ventricular ejection fraction
